# Primary Vitamin D Target Genes Allow a Categorization of Possible Benefits of Vitamin D_3_ Supplementation

**DOI:** 10.1371/journal.pone.0071042

**Published:** 2013-07-29

**Authors:** Carsten Carlberg, Sabine Seuter, Vanessa D. F. de Mello, Ursula Schwab, Sari Voutilainen, Kari Pulkki, Tarja Nurmi, Jyrki Virtanen, Tomi-Pekka Tuomainen, Matti Uusitupa

**Affiliations:** 1 School of Medicine, Institute of Biomedicine, University of Eastern Finland, Kuopio, Finland; 2 Institute of Public Health and Clinical Nutrition, Department of Clinical Nutrition, University of Eastern Finland, Kuopio, Finland; 3 Institute of Clinical Medicine, Internal Medicine Kuopio University Hospital, Kuopio, Finland; 4 Eastern Finland Laboratory Centre and Department of Clinical Chemistry, University of Eastern Finland, Kuopio, Finland; 5 Institute of Public Health and Clinical Nutrition, Department of Public Health, University of Eastern Finland, Kuopio, Finland; 6 Research Unit, Kuopio University Hospital, Kuopio, Finland; Roswell Park Cancer Institute, United States of America

## Abstract

Vitamin D deficiency has been associated with an increased risk of developing a number of diseases. Here we investigated samples from 71 pre-diabetic individuals of the VitDmet study, a 5-month high dose vitamin D_3_ intervention trial during Finnish winter, for their changes in serum 25-hydroxyvitamin D_3_ (25(OH)D_3_) concentrations and the expression of primary vitamin D target genes in peripheral blood mononuclear cells and adipose tissue. A negative correlation between serum concentrations of parathyroid hormone and 25(OH)D_3_ suggested an overall normal physiological vitamin D response among the participants. The genes *CD14* and thrombomodulin (*THBD*) are up-regulated primary vitamin D targets and showed to be suitable gene expression markers for vitamin D signaling in both primary tissues. However, in a ranking of the samples concerning their expected response to vitamin D only the top half showed a positive correlation between the changes of *CD14* or *THBD* mRNA and serum 25(OH)D_3_ concentrations. Interestingly, this categorization allows unmasking a negative correlation between changes in serum concentrations of 25(OH)D_3_ and the inflammation marker interleukin 6. We propose the genes *CD14* and *THBD* as transcriptomic biomarkers, from which the effects of a vitamin D_3_ supplementation can be evaluated. These biomarkers allow the classification of subjects into those, who might benefit from a vitamin D_3_ supplementation, and others who do not.

## Introduction

Vitamin D is a fat-soluble vitamin that can be obtained by diet but is mainly synthesized from 7-dehydrocholesterol in an UV-B-dependent reaction in the skin [1]. In the liver, vitamin D_3_ is then further converted into 25-hydroxyvitamin D_3_ (25(OH)D_3_), which is the major storage form of vitamin D. In the kidney, 25(OH)D_3_ is further 1α-hydroxylated to yield its hormonal form, 1,25-dihydroxyvitamin D_3_ (1,25(OH)_2_D_3_), which acts as a ligand to the transcription factor vitamin D receptor (VDR) [2]–[4]. Serum 25(OH)D_3_ concentration is the widely accepted indicator of the vitamin D status for the human body [5]. Vitamin D deficiency is defined as a serum 25(OH)D_3_ concentration of below 50 nM (20 ng/ml) [6] and affects more than 1 billion humans, i.e. it is one of most common health risks [7]. Vitamin D deficiency is caused by the lack of adequate vitamin D in diet and an insufficient exposure to sun [8]. The musculoskeletal consequences of inadequate vitamin D concentrations are well established and include rickets in children and osteomalacia and fractures in adults [9]. A growing number of other diseases, such as type 1 and type 2 diabetes, cardiovascular disease and cancers of the breast, prostate and colon, have also been linked to vitamin D insufficiency, but causal associations have not yet been fully established [10]–[14]. In contrast, high vitamin D concentrations can cause overcalcification of bones, soft tissues, heart and kidneys leading to kidney stones and hypertension [15], but these side effects occur only at 25(OH)D_3_ concentrations above 240 nM [16].

The classical, physiological role of 1,25(OH)_2_D_3_ and its receptor is the regulation of calcium and phosphate homeostasis and bone mineralization [17], but there is also a lot of evidence that VDR ligands have anti-proliferative and immuno-modulatory functions [18], [19]. This fits both with the widespread expression of the VDR and the above described consequences of vitamin D deficiency. Transcriptome-wide analysis indicated that per cell type between 200 and 600 genes are primary targets of vitamin D [20]–[23]. Since most of these genes respond to vitamin D in a cell-specific fashion, the total number of vitamin D targets in the human genome is far higher than 1,000. This is supported by the genome-wide view on VDR binding sites in human lymphoblastoids [20], THP-1 human monocytic leukemia cells [21] and LS180 human colorectal cancer cells [24] obtained by chromatin immunoprecipitation combined with high throughput sequencing (ChIP-seq). These studies reported between 1,600 and 2,800 genomic VDR binding sites that locate both up- and downstream of the transcription start sites of primary vitamin D target genes. However, the three ChIP-seq datasets overlap only at some 200 genomic locations [25], i.e. there is a limited set of conserved targets of vitamin D and its receptor also on the genomic level.

Sufficient exposure to natural UV-B radiation or adequate intake from diet or supplements is needed to achieve an optimal serum 25(OH)D_3_ concentration. However, the change in serum 25(OH)D_3_ concentrations can vary widely from person to person. Diet and sun exposure together with age and adiposity in average account only for some 30% of the inter-individual variation in 25(OH)D_3_ serum concentrations [26]. Accordingly, genetic and epigenetic factors are responsible for the main variation in vitamin D concentrations [27]–[29]. Based on this wide inter-individual response variation, it is obvious that a “one-size-fits-all” approach will not work ideally for vitamin D supplementation. Therefore, we investigated in this study samples from 71 pre-diabetic participants of the VitDmet cohort, a 5-month high dose vitamin D_3_ intervention trial during Finnish winter [30], for their changes in serum 25(OH)D_3_ concentrations and the expression of primary vitamin D target genes in peripheral blood mononuclear cells and adipose tissue. Only the top half of a ranking concerning response to vitamin D provided a significant correlation between the changes of *CD14* or *THBD* mRNA and serum 25(OH)D_3_ concentrations. We present *CD14* and *THBD* as transcriptomic biomarkers, from which straightforward conclusions on the benefits of a vitamin D_3_ supplementation can be obtained.

## Materials and Methods

### VitDmet study design and ethics statement

Participants donating PBMCs and adipose tissue samples had been participating in the VitDmet study (NCT01479933, ClinicalTrials.gov). They were selected to be ≥60 years of age for males or ≥65 for females, showed evidence of disturbed glucose homeostasis, i.e. impaired fasting glucose or impaired glucose tolerance, but no type 2 diabetes, and had a body mass index (BMI) between 25 and 35. An additional inclusion criterion was a baseline serum 25(OH)D_3_ concentration of below 75 nM [30]. The research ethics committee of the Northern Savo Hospital District had approved the study protocol. All participants gave a written informed consent to participate in the study.

### Serum measurements

Serum 25(OH)D_3_ concentrations were measured from venous blood samples using a high performance liquid chromatography with coulometric electrode array as described previously [31]. Only a few study participants had a quantifiable serum concentration of 25(OH)D_2_, which is of plant origin; therefore only 25(OH)D_3_ was considered. Serum concentrations of PTH were assayed by a chemiluminescence kit (DiaSorin, Dietzenbach, Germany) and those of IL6 by ELISA (Quantikine Kit; R&D Systems, Minneapolis, MN, USA). Serum calcium was determined with colorimetric Arsenazo III test using a Konelab 20XT clinical chemistry analyzer (Thermo Fisher Scientific).

### PBMC isolation, adipose tissue biopsy and sample preparation

Blood samples for PBMC isolation and adipose tissue samples were collected after overnight (12 h) fasting of the study participants. PBMCs were isolated from 8 ml of peripheral blood in a Vacutainer CPT Cell Preparation Tube with sodium citrate (BD, Franklin Lakes, NJ, USA) according to the manufacturer's instructions and stored at −80°C until used for RNA extraction [32]. Adipose tissue (0.5–1 g) was taken by needle biopsy from subcutaneous abdominal adipose tissue under local anesthesia (10 mg/ml lidocaine without adrenalin). The adipose tissue samples were washed twice with phosphate-buffered saline, frozen quickly in liquid nitrogen and stored at −80°C until used for RNA extraction.

### RNA extraction and cDNA synthesis

Total RNA from PBMCs and adipose tissue samples was extracted using the TRIzol method followed by further purification with miRNeasy Mini Kit columns according to the instructions provided by the manufacturer (Qiagen). The RNA concentration and the A260/A280 ratio were measured using the NanoDrop spectrophotometer, an acceptable ratio being 1.9–2.1 [33]. The total RNA was reverse transcribed into cDNA using the High-Capacity cDNA Archive Kit (Applied Biosystems).

### Quantitative PCR (qPCR)

qPCR reactions were performed using 250 nM of reverse and forward primers, 2 µl cDNA template (25 ng RNA/µl in the cDNA synthesis reaction and then diluted 1/10 prior to the PCR reaction) and the Roche LightCycler 480 SYBRGreen I Master (Roche) in a total volume of 10 µl. In the PCR reaction the hotstart Taq polymerase was activated for 10 min at 95°C, followed by 42 amplification cycles of 20 s denaturation at 95°C, 15 s annealing at primer-specific temperatures (Table S1 in File S1) and 15 s elongation at 72°C and a final elongation for 10 min at 72°C. PCR product specificity was monitored using post-PCR melt curve analysis. Relative expression levels were determined with the comparative delta threshold cycle (delta-Ct) method. Relative expression levels of the target genes were normalized to the internal reference genes *B2M*, *GAPDH* and *HPRT1* (PBMCs) or *GAPDH* and *RPLP0* (adipose tissue) using the geNorm algorithm [34]. Briefly, the arithmetic mean of replicated Ct values for each gene is transformed to a relative quantity (setting the sample with the highest expression as calibrator to 1), using the delta-Ct formula Q  =  E^deltaCt^  =  E^(calibratorCt – sampleCt)^ (Q  =  quantity sample relative to calibrator sample; E  =  amplification efficiency). For normalization, the relative quantities were divided by the normalization factor being the geometric mean of the reference genes.

### Data analysis

Linear regression analysis was performed using StatPlus^®^ software, version 2009 (AnalystSoft, Alexandria, VA, USA). P-values were determined by analysis of variance (ANOVA). The ranking of vitamin D responsiveness was done under the assumption of a linear positive correlation between changes of the mRNA expression of *CD14* or *THBD* and changes in the 25(OH)D_3_ serum concentrations. For each tissue and gene the samples were ranked symmetrically to the median of the sorted ratios of gene expression and 25(OH)D_3_ serum concentration changes. The mean of these four lists determined the final ranking, taking into account that for 24 participants no adipose tissue samples were available.

## Results

### Characteristics of the VitDmet cohort

With the aim to investigate possible effects of vitamin D_3_ supplementation on glucose metabolism and other features of the metabolic syndrome, a three-armed trial (placebo, 40 or 80 µg daily vitamin D_3_ supplementation over 5 months) had been designed. The study recruited 73 pre-diabetic (impaired fasting glucose or impaired glucose tolerance) human subjects from the region of Kuopio, Finland (63 °N) [30]. The trial started in October and finished at the end of winter (April), i.e. in a season of the year where in the Northern hemisphere at this latitude there is no natural UV-B source to induce vitamin D_3_ synthesis in the skin of the participants.

In the present study, we focused on a sub-group of 71 participants of the above-described cohort, for which PBMC isolates were available from both the start and the end of the trial. For 47 of these 71 individuals also subcutaneous adipose tissue biopsies had been taken at both time points. The basic clinical and biochemical variables of the participants (Table 1) showed that neither body weight nor BMI changed significantly during the intervention in any of its arms. Importantly, in none of the three groups a significant change in serum calcium concentrations was observed, although one group obtained the rather high dose of 80 µg vitamin D_3_/day. The baseline serum 25(OH)D_3_ concentrations of the 71 individuals ranged between 35.9 and 73.6 nM with an average of 58.6 nM (Tables 1 and Table S2 in File S2). During the intervention the 25(OH)D_3_ serum concentrations raised in average by 24.9 nM, but only by 1.1 nM in the control group, by 26.7 nM in the group that was supplemented by 40 µg vitamin D_3_ per day and by 44.9 nM in the group that received the highest supplementation dose of 80 µg (p<0.001 across the groups). Accordingly, serum PTH concentrations decreased in the groups with 40 and 80 µg daily vitamin D_3_ doses as compared to the placebo group.

**Table 1:** Main clinical and biochemical characteristics of the VitDmet study participants at baseline and their changes during the study.

Parameter	Placebo group*, n = 22**	40 µg vitamin D_3_/day group, n = 25	80 µg vitamin D_3_/day group, n = 24
**Serum [25(OH)D_3_] start (nM)**	58.9+/−10.2	59.0+/−7.6	57.8+/−10.3
**Delta serum [25(OH)D_3_] (nM)**	1.1 (−4.7; 6.9)	26.7 (20.0; 33.4)	44.8 (36.2; 53.4)
**BMI start (kg/m^2^)**	30.2+/−2.8	28.8+/−2.7	29.5+/−3.0
**Delta BMI (kg/m^2^)**	0.23 (−0.12; 0.58)	0.34 (0.13; 0.55)	0.33 (0.07; 0.53)
**Serum [PTH] start (pg/ml)**	44.6+/−18.2	41.5+/−9.5	43.8+/−11.1
**Delta serum [PTH] (pg/ml)**	4.7 (1.4; 8.0)	−0.5 (−3.5; 2.5)	−3.7 (−6.1; −1.3)
**Serum [Ca] start (mM)**	2.35+/−0.09	2.31+/−0.05	2.33+/−0.08
**Delta serum [Ca] (mM)**	−0.06 (−0.09; −0.03)	−0.04 (−0.07; −0.01)	−0.03 (−0.06; 0.00)
**Age (years)**	67.4+/−5.7	66.2+/−5.5	66.4+/−4.3
**Gender (female/male)**	3 (13.6%)/19 (86.4%)	4 (16%)/21 (84%)	3 (12%)/21 (88%)

* all participants were asked to keep their diet and other lifestyle habits unchanged during the study and were allowed to take up to 20 µg vitamin D_3_/day.

** random assignment of the participants to the three groups.

Means and standard deviations for the baseline characteristics before intervention or mean parameter change with 95% confidence interval after the intervention are indicated for the three arms of the study. The detailed data of all 71 study participants are shown in Table S2 in File S2.

The analysis of the individual participants (Table S2 in in File S2) showed that the serum 25(OH)D_3_ concentrations of 11 persons decreased as much as 30.2 nM, mostly in the placebo group, while 25(OH)D_3_ raised in the remaining participants by up to 87.2 nM. Taking the Institute of Medicine's (IoM) recommended 25(OH)D_3_ serum concentration of 50 nM as a reference [6], at the start of the trial 14 individuals (19.7%) had been vitamin D deficient, while at its end only 5 persons (7%) had low 25(OH)D_3_ concentrations. In contrast, based on the alternatively discussed threshold of 75 nM [35], which was also applied as an inclusion criterion in the present study, at entry all participants (100%) had insufficient vitamin D concentrations, while after the intervention this applied only to 29 persons (40.8%), 18 of which were in the not-supplemented placebo control group. However, the variations in the serum 25(OH)D_3_ concentrations were not fully consistent with the vitamin D_3_ supplementation, which in part can be explained by the fact that all participants were allowed to continue their own vitamin D_3_ supplementation with a lower dose (up to 20 µg/day). As we found no meaningful correlation of VDR target gene expression between the original study groups, for the following we ignored the information for the type of vitamin D_3_ supplementation and took for each of the 71 participants only the relative changes in serum 25(OH)D_3_ concentrations into account.

In summary, the gene expression analysis of this study is based on PBMC and adipose tissue samples, which were obtained from a cohort of 71 pre-diabetic elderly participants with a wide range of change in the 25(OH)D_3_ serum concentrations after a 5-month vitamin D_3_ supplementation during Finnish winter.

### Gene expression characteristics of the study participants

The *PTH* gene is a well-known down-regulated primary target of vitamin D and its receptor, while PTH protein signaling up-regulates *CYP27B1* gene expression [36], [37]. This inverse correlation between vitamin D and PTH concentrations is of major importance for appropriate bone mineralization [38]. In this study, we could confirm this inverse correlation, when plotting the relative change of PTH concentrations against the change of the serum 25(OH)D_3_ concentrations (R^2^  = 0.231, p = 0.00002; Fig. S1 in File S1). This indicates that the overall vitamin D responsiveness of the 71 participants of the study is according to expectations.

**Figure 1:**
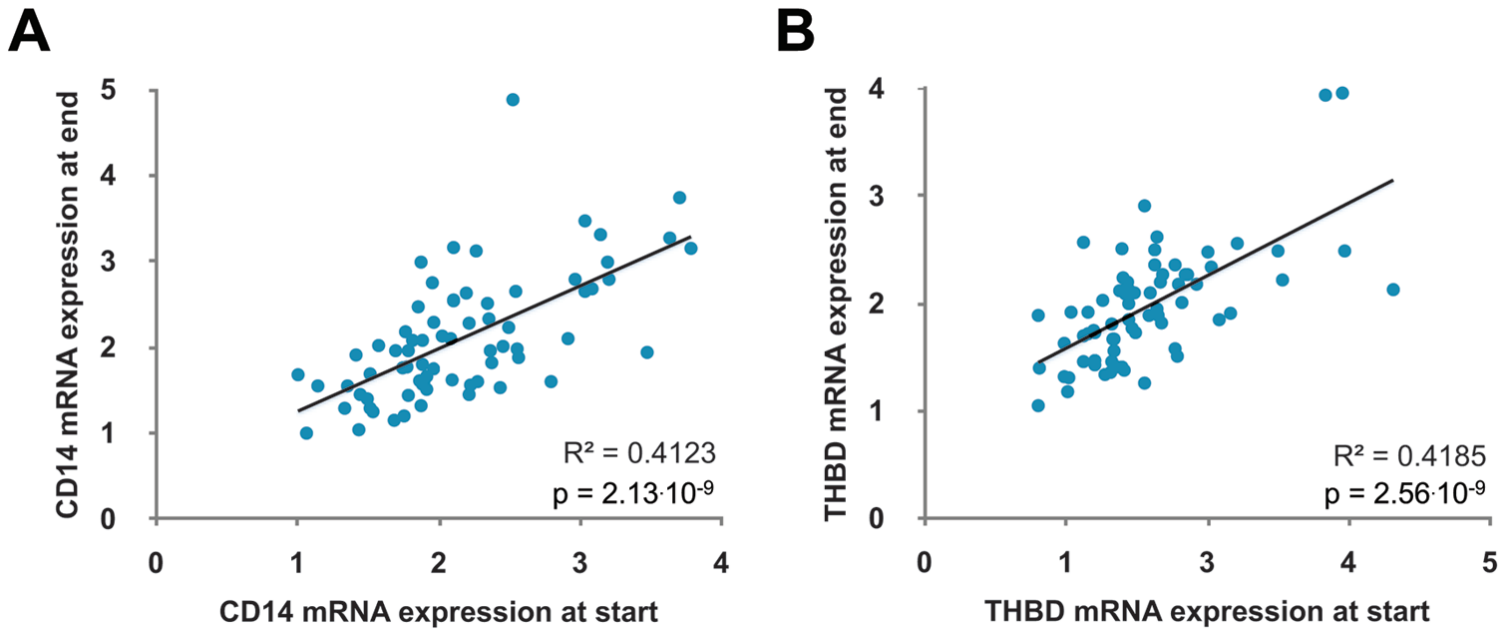
The genes *CD14* and *THBD* are suitable markers for vitamin D signaling in PBMCs. For all 71 participants the mRNA expression in PBMCs of the genes *CD14* (A) and *THBD* (B) at the start of the study correlate positively with their expression at the end.

Since the *PTH* gene is exclusively expressed in the parathyroid gland, i.e. in a tissue, which is not easily available for biopsies, it is not an appropriate choice for vitamin D gene expression studies. Therefore, we screened for more globally expressed primary VDR target genes. Our transcriptome- and genome-wide studies in THP-1 monocytes [21], [39] suggested the genes *CD14* and *THBD* to be well suited. In contrast to many other primary VDR target genes, *CD14* and *THBD* are known for their prominent, long-lasting response to treatment with 1,25(OH)_2_D_3_. Moreover, both genes are regulated by a strong VDR binding site, which for *CD14* is conserved between VDR ChIP-seq datasets of monocytes and colon cells (Fig. S2A in File S1) and for *THBD* even between monocytes, lymphoblastoids and colon cells (Fig. S2B in File S1). Interestingly, in a very recent study the same two genes were found in a comparison of a larger number of vitamin D transcriptomics studies (including our own) to be most significantly correlated with serum 25(OH)D_3_ concentrations of the Norwegian Women and Cancer Post-genome cohort [40]. Accordingly, we found that the relative mRNA expression of *CD14* and *THBD* in PBMCs (normalized by three housekeeping genes) isolated from the 71 participants at the start of the study correlated highly significantly with the respective expression in cells taken at the end of the intervention (R^2^  = 0.4123, p = 3.13.10^−9^ and R^2^  = 0.4185, p = 2.56.10^−9^; Figs. 1A and B). This correlation came out to be even better, when the *CD14* and *THBD* mRNA expression levels were further normalized by their respective *VDR* mRNA levels (R^2^  = 0.6709, p = 2.49.10^−11^ and R^2^  = 0.7428, p = 1.88.10^−15^; Figs. S3A and B in File S1).

**Figure 2:**
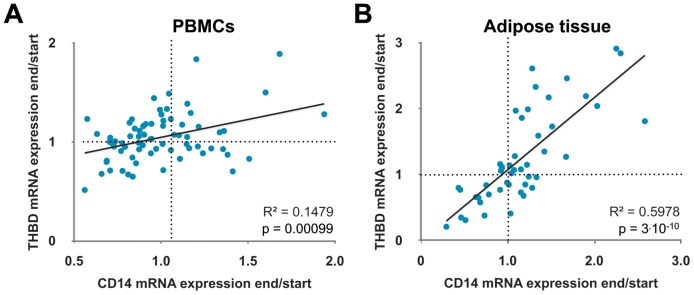
The genes *CD14* and *THBD* correlate to each other in their expression. In PBMCs (A) and adipose tissue (B) the ratios of the mRNA expression of *CD14* at the end and the start of the study also positively correlate with the respective ratios of *THBD* mRNA expression.

For all 71 individuals the change of mRNA expression levels of *CD14* and *THBD* positively correlated to each other (R^2^  = 0.1479, p = 0.00099; Fig. 2A). Interestingly, an even far more significant correlation between the changes of *CD14* and *THBD* mRNA expression levels was observed in adipose tissue samples obtained from 47 of the study participants (R^2^  = 0.5978, p = 3.10^−10^; Fig. 2B).

Taken together, the expected negative correlation between serum concentrations of PTH and 25(OH)D_3_ confirmed an overall normal physiological vitamin D response of the 71 study participants. The primary VDR target genes *CD14* and *THBD* showed to be suitable transcriptomic biomarkers of vitamin D signaling both in PBMCs and adipocytes.

### *CD14* and *THBD* mRNA expression changes efficiently classify study participants

Based on the proven overall functionality of vitamin D signaling in the tissue samples of the study participants we assumed that an increase in serum 25(OH)D_3_ concentrations would result in a respective increase in *CD14* and *THBD* mRNA expression. However, neither with all 71 PBMCs donors nor with the subgroup of all 47 adipose tissue donors we could observe any significant correlation between the changes in 25(OH)D_3_ serum concentrations and the mRNA expression changes of *CD14* or *THBD* in the respective tissue samples (data not shown). Based on the assumption of a linear positive correlation between the changes of *CD14* or *THBD* gene expression both in PBMCs and adipose tissue and changes of 25(OH)D_3_ serum concentrations, we performed four separate rankings of the study participants, which were then combined to one ranking (Table S2 in File S2).

**Figure 3:**
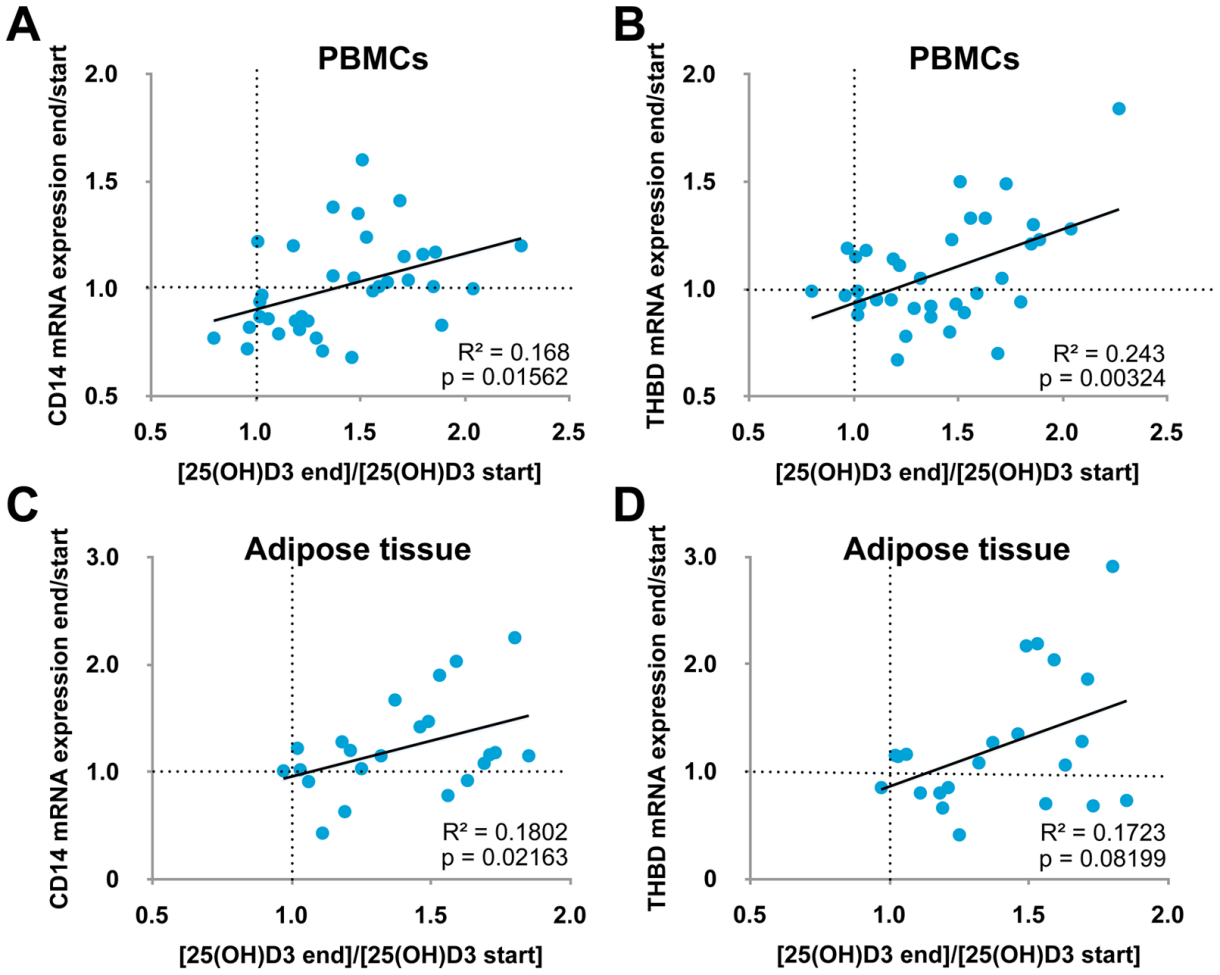
Changes of 25(OH)D_3_ serum concentrations correlate with gene expression in PBMCs and adipocytes. The study participants were ranked for their changes of *CD14* and *THBD* mRNA expression in PBMCs and adipocytes. For the top half of the PBMC donors (35 individuals, A and B) and also for the top half of the adipose tissue donors (23 åindividuals, C and D) changes of the mRNA expression of *CD14* (A, C) and *THBD* (B, D) from start to end of the study positively correlate with the respective change of 25(OH)D_3_ serum concentrations.

When we took only the top half of the ranking (35 persons), the plot of the changes in *CD14* (Fig. 3A) or *THBD* (Fig. 3B) mRNA expression levels in PBMCs against the respective changes in 25(OH)D_3_ concentrations provided statistically significant positive correlations (R^2^  = 0.168, p = 0.01562 and R^2^  = 0.243, p = 0.00324). We could confirm this observation with the adipose tissue samples, when restricting to the top half of the ranking in this tissue (23 persons). However, in adipose tissue the positive correlation between the changes in *CD14* (R^2^  = 0.1802, p = 0.02163; Fig. 3C) or *THBD* (R^2^  = 0.1723, p = 0.08199; Fig. 3D) mRNA expression levels and their respective 25(OH)D_3_ serum concentrations is less significant than in PBMCs. As a negative control for VDR target gene specificity, in neither of the two tissues the changes in *VDR* mRNA expression correlated with 25(OH)D_3_ serum concentration changes (data not shown).

**Figure 4:**
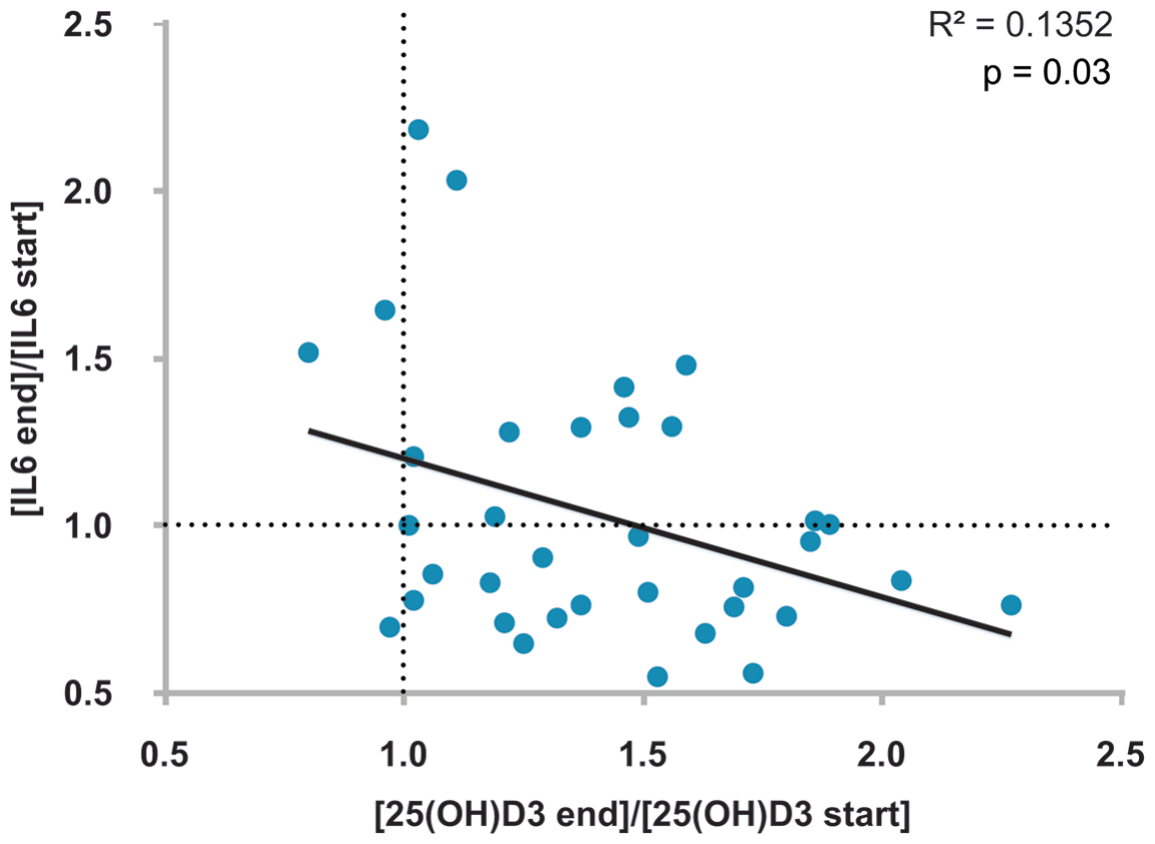
Negative correlation of serum concentrations for 25(OH)D_3_ and IL6 protein. For the top half of the individuals that were ranked based on their vitamin D responsiveness the ratio of the 25(OH)D_3_ serum concentrations at the end and the start of the study is plotted against the respective change of the IL6 protein concentrations.

The categorization of the study participants by response to a change in their 25(OH)D_3_ serum concentrations allows to observe correlations that are not visible when all participants are included in the analyses. For example, on the basis of all 71 participants there is no correlation between the change in serum concentrations of 25(OH)D_3_ and the inflammatory marker IL6 [41] (data not shown). However, when we analyzed the segregated set of the 35 most vitamin D responsive samples, we found a significant negative correlation between changes in 25(OH)D_3_ and IL6 (R^2^  = 0.1352, p = 0.03; Fig. 4), as described earlier for monocytes [42].

In summary, the changes of *CD14* or *THBD* mRNA expression levels in PBMCs and in adipose tissue samples in relation to 25(OH)D_3_ serum concentrations suggest that it is possible to identify subjects who may benefit from vitamin D_3_ supplementation, for example manifested by the down-regulation of the inflammatory marker serum IL6.

## Discussion

During the last years a large number of population-based studies initiated a remarkable re-evaluation of the benefits of vitamin D for human health. This had been further catalyzed by the discussion on the recommendation of the IoM concerning the sufficient serum 25(OH)D_3_ concentration of 50 nM [6]. Moreover, vitamin D belongs to the few nutritional compounds, which has, via its metabolite 1,25(OH)_2_D_3_, a direct effect on gene regulation [3]. This property allows the discovery and characterization of genomic and transcriptomic biomarkers that enable a direct evaluation of both beneficial and possible unfavorable effects of vitamin D. In the present study, we tested our hypothesis that some primary vitamin D target genes may serve as such biomarkers. We suggest that measuring the change in mRNA expression of the genes *CD14* and *THBD* in response to vitamin D_3_ supplementation allows the evaluation of the responsiveness of the vitamin D signaling system of the human individual and provides a guideline if and how to adjust his/her serum vitamin D concentrations.

The PBMC and adipose tissue samples used in this study have the advantage of originating from a rather long vitamin D_3_ intervention trial (5 months) with a placebo and a quite high dose of supplementation (up to 80 µg/day, i.e. 3,200 IU). The study was performed in the dark season of the year (Finnish winter) where there is no natural UV-B-induced endogenous production of vitamin D_3_. This design resulted at the end of the study in i) a large range of serum 25(OH)D_3_ concentrations between 27.5 and 155.7 nM and ii) changes ranging from a decrease by 30.2 nM and an increase by 87.2 nM. In contrast to most other studies reported, we express these changes in relative and not in absolute terms, i.e. as a ratio and not as a difference. Therefore, we interpret the changes in serum 25(OH)D_3_ concentrations, which were achieved by the VitDmet study, as a range from a 2.1-fold decrease of the baseline levels up to a 2.8-fold increase. In this way, our approach is closer to the analysis of a typical ligand stimulation experiment as it is the standard in mechanistic studies [39]. Accordingly, the mRNA expression changes range in PBMCs from a 1.8-fold decrease to a 1.9-fold increase for *CD14*, from a 2.0-fold decrease to a 1.9-fold increase for *THBD* and from a 1.8-fold decrease to a 1.9-fold increase for *VDR*. In adipose tissue samples the ranges in mRNA expression changes are even larger spanning from a 3.4-fold decrease to a 2.6-fold increase for *CD14*, from a 4.8-fold decrease to a 2.9-fold increase for *THBD* and from a 4.0-fold decrease to a 4.1-fold increase for *VDR*. Interestingly, although *VDR* expression changes do not correlate with changes in 25(OH)D_3_ serum concentrations, the *VDR* gene shows similar ranges of variation than *CD14* and *THBD*.

Although the ranges of the 25(OH)D_3_ serum concentration and VDR target gene changes during the intervention are in the same order, there is no statistically significant correlation between them, when all 71 study participants are studied. However, after ranking the study participants by the responsiveness of their *CD14* and *THBD* expression to changes of 25(OH)D_3_ concentrations in both tested tissues, we found in the top half of the ranked participants a significant positive correlation. From the latter 35 individuals only 3 showed a slight decrease in 25(OH)D_3_ concentrations, i.e. majority of them seem to benefit from the intervention irrespective of their initial serum 25(OH)D_3_ concentration. In fact, only 4 of the 35 participants had an initial 25(OH)D_3_ concentration of below 50 nM, i.e. according to the recent IoM recommendations [6] most of the participants would not have needed a vitamin D supplementation. For the other half of the study group no relationship between changes in 25(OH)D_3_ concentrations and VDR target gene expression could be found. These individuals showed a more individual response to vitamin D supplementation (or the lack of it) and no general conclusion could be reached from gene expression data.

We suggest that analysis of the responsiveness of the genes *CD14* and *THBD* to changes in 25(OH)D_3_ serum concentrations allows a categorization of the study participants. Half of the participants can be considered as conventional responders to vitamin D. These individuals have a fully functional vitamin D signaling system and their vitamin D concentrations have not reached saturation. This is proven by the down-regulation of IL6 protein in serum. IL6 has not yet been shown to be a primary VDR target, but it is known as one of the genes, via which the anti-inflammatory effect of vitamin D is mediated [42], [43]. IL6 is a marker of low-grade inflammation and has been suggested as a risk factor for type 2 diabetes [44] and cardiovascular disease [45]. Therefore, the down-regulation of IL6 protein in response to raising 25(OH)D_3_ serum concentrations is an indication of a beneficial effect of vitamin D_3_ supplementation.

The persons in the lower ranking half may have either i) one or the other restriction in their response to vitamin D or ii) a superefficient response to vitamin D, so that they are already saturated with lower levels of circulating vitamin D. The genetic basis for baseline 25(OH)D_3_ concentrations are SNPs in genes encoding for vitamin D metabolizing enzymes, such as *CYP2R1* and *CYP24A1*, in the vitamin D-binding protein (encoded by the gene *GC*) and in the *VDR* gene [46], [47]. Moreover, the overall efficiency of vitamin D signaling is very likely based on a number of so far largely non-characterized polymorphisms in genomic VDR binding sites [20]. Further studies are needed to stratify individuals as to their susceptibility to vitamin D deficiency. In such studies the individual's response to vitamin D_3_ supplementation will be correlated with his/her genomic profile of VDR binding sites.

In a very recent report the gene expression patterns of eight healthy adults were followed, which had been supplemented daily with either 10 or 40 µg vitamin D_3_ in the Boston area (42 °N) for 2 months during winter time [23]. It describes close to 300 genes to be more than 1.5-fold changed in expression and the genes *CD83*, *TNFAIP3*, *KLF10* and *SBDS* were selected as most representative markers. We found that only the *KLF10* gene shows in colon cancer cells a genomic VDR binding site, i.e. there is not much evidence that these four markers genes are primary VDR targets with a global expression profile as reported here for *CD14* and *THBD*. Therefore, the expression changes of the four described genes probably cannot be used for a similar type of categorization as performed in this study with *CD14* and *THBD*.

In conclusion, we suggest that the genes *CD14* and *THBD* are suitable biomarkers for displaying the transcriptomic response of human tissues to vitamin D_3_ supplementation. However, only half of all investigated persons seem to respond in a way that we expected based on principles of vitamin D gene regulation to changes in their serum 25(OH)D_3_ concentrations, granting that from the response of their *CD14* and *THBD* mRNA expressions straightforward conclusions on the benefits of a vitamin D_3_ supplementation can be obtained. This suggests a categorization of the study participants into those that are regular responders to vitamin D and others with an irregular response. The latter persons may have either some variation in their vitamin D signaling, which make more complex investigations necessary, or they simply have a sufficient vitamin D concentration, so that further supplementation is unnecessary.

## Supporting Information

File S1 (PDF)

File S2 (XLSX)
