# Supplementary material for: Primary Vitamin D Target Genes Allow a Categorization of Possible Benefits of Vitamin D3 Supplementation
Source: PLoS One. 2013 Jul 29;8(7):e71042. doi: 10.1371/journal.pone.0071042 (PMC3726591; doi:10.1371/journal.pone.0071042)
Supplement: File S1 — (PDF) [file pone.0071042.s001.pdf]

## SUPPLEMENTARY MATERIAL

## SUPPLEMENTARY TABLES

### Table S1: qPCR primers.

| **Gene** | **Fragment size (bp)** | **Annealing temperature (°C)** | **Primer sequences (5'-3')** |
|---|---|---|---|
| ***B2M*** | 246 | 60 | GGCTATCCAGCGTACTCCAAA<br>CGGCAGGCATACTCATCTTTTT |
| ***CD14*** | 142 | 60 | ACGCCAGAACCTTGTGAGC<br>GCATGGATCTCCACCTCTACTG |
| ***GAPDH*** | 113 | 60 | CATGAGAAGTATGACAACAGCC<br>AGTCCTTCCACGATACCAAAGT |
| ***HPRTI*** | 94 | 60 | TGACACTGGCAAAACAATGCA<br>GGTCCTTTTCACCAGCAAGCT |
| ***RPLP0*** | 318 | 60 | AGATGCAGCAGATCCGCAT<br>GTGGTGATACCTAAAGCCTG |
| ***THBD*** | 107 | 60 | GACCTTCCTCAATGCCAGTCA<br>CGTCGCCGTTCAGTAGCAA |
| ***VDR*** | 332 | 60 | AGATGACCCTTCTGTGACCC<br>AGCTTCTTCAGTCCCACCTG |

**Table S2: Ranking of vitamin D response.** Complete data set for 71 participants that donated PBMCs. From 47 of them also adipose tissue biopsies were taken. Ranking is based on response of both tissues to vitamin D.

## SUPLEMENTARY FIGURE LEGENDS

**Figure S1: Negative correlation of serum concentrations for 25(OH)$D_3$ and PTH protein.** For all 71 participants the ratio of the 25(OH)$D_3$ concentration at the end and the start of the study is plotted against the respective change of the PTH serum concentrations.

**Figure S2: VDR binding regions of the genes *CD14* and *THBD*.** VDR ChIP-seq data for the genomic loci of the genes *CD14* (A) and *THBD* (B) are shown. The peak tracks show data from LS-180 colon carcinoma cells (pink [24]), lymphoblastoids (light blue [20]) and THP-1 monocytes (red [21]) comparing genomic VDR binding in unstimulated or vehicle-stimulated cells with that after 1,25$(OH)_2D_3$ (1,25D) treatment for indicated times. The structure of the genes is shown in blue and the respective VDR peaks are boxed.

**Figure S3: *CD14* and *THBD* expression levels normalized by VDR expression.** The mRNA expression of the genes *CD14* (A) and *THBD* (B) in PBMCs at the end and the start of the study correlate even better, when it is normalized bymRNA expression.

# Fig. S1

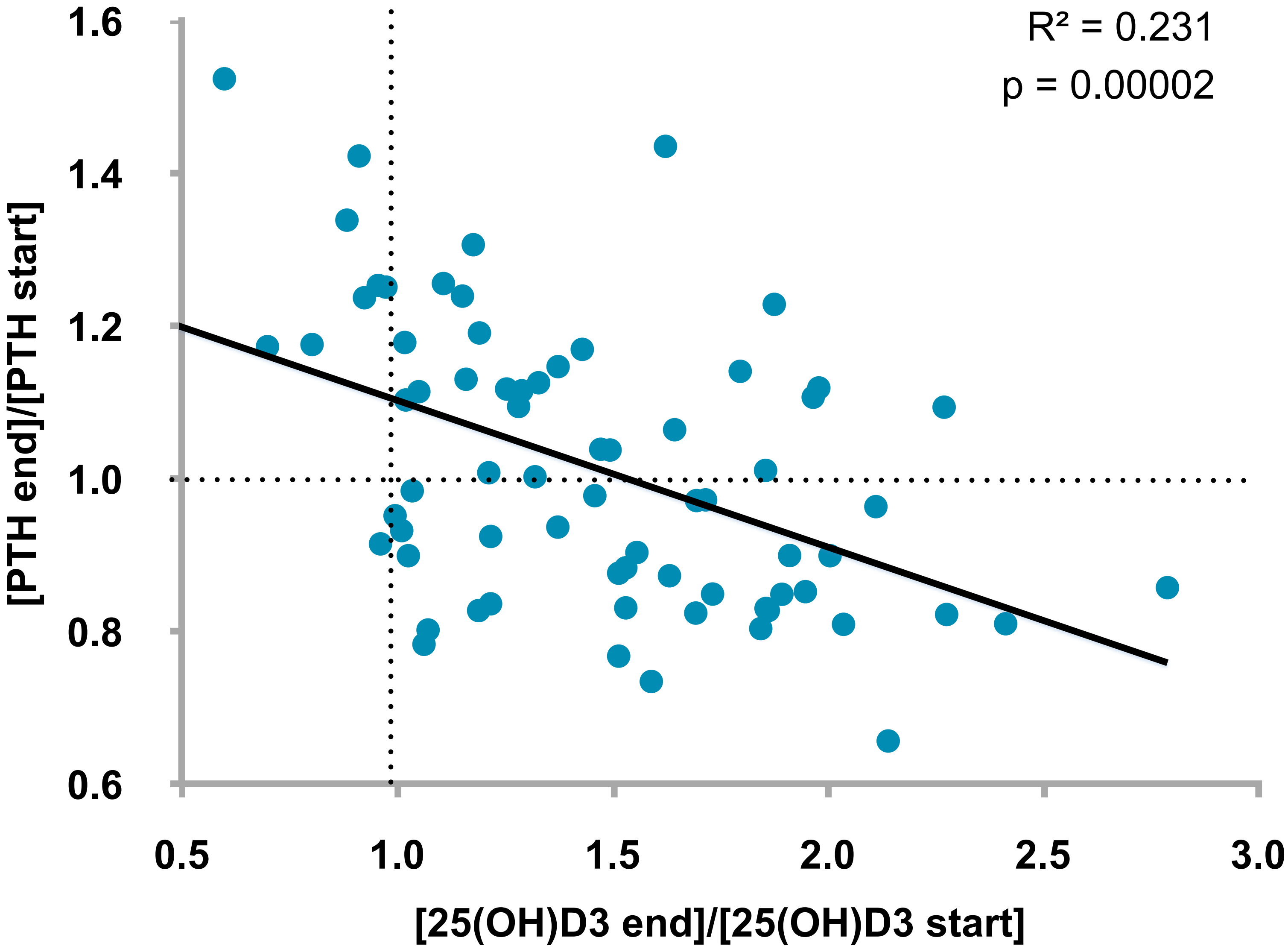
R² = 0.231
p = 0.00002
1.6
1.4
1.2
1.0
0.8
0.6
[PTH end]/[PTH start]
0.5
1.0
1.5
2.0
2.5
3.0
[25(OH)D3 end]/[25(OH)D3 start]

# Fig. S2

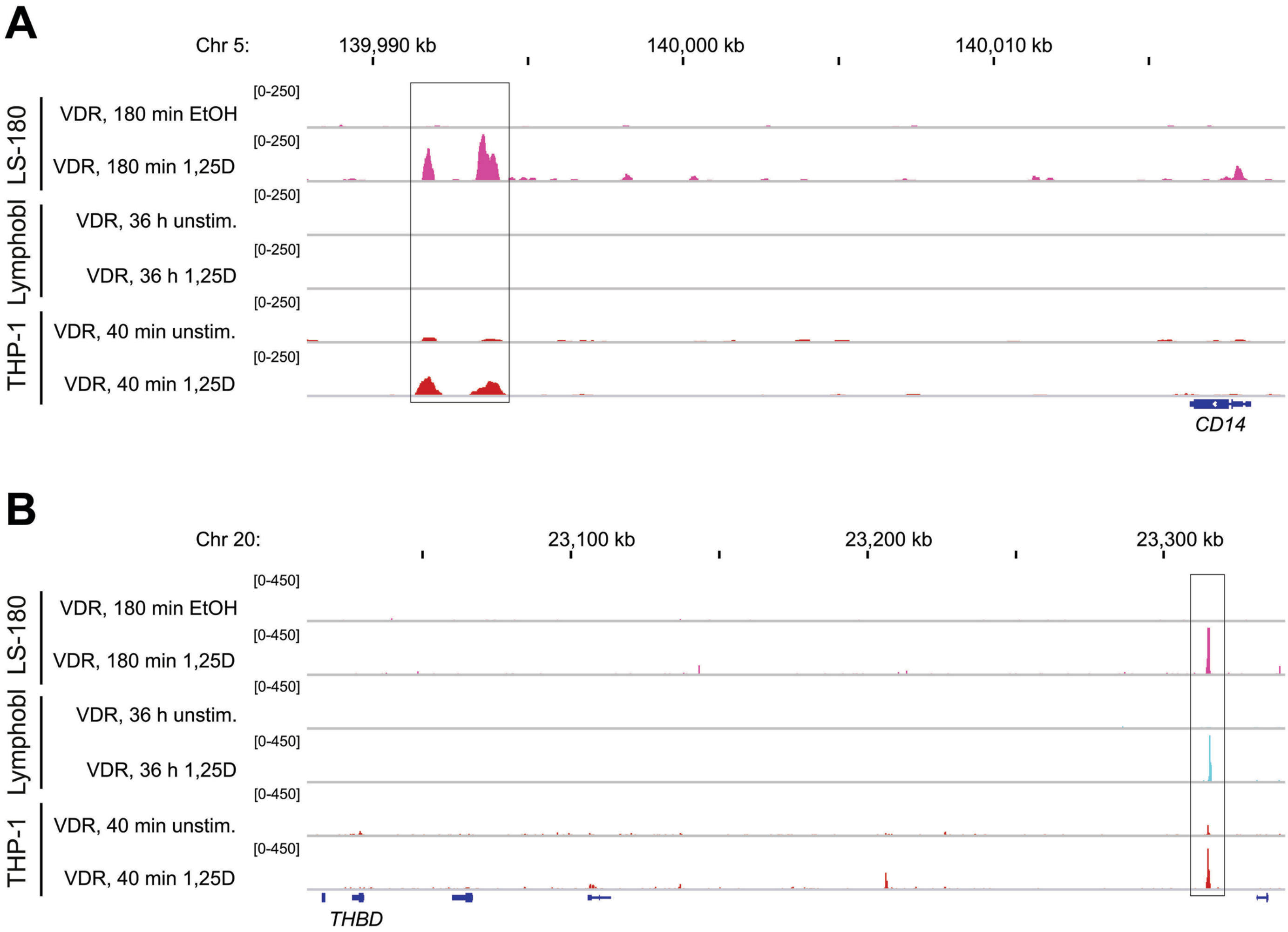
A
Chr 5:
139,990 kb
140,000 kb
140,010 kb
[0-250]
LS-180
VDR, 180 min EtOH
VDR, 180 min 1,25D
Lymphobl
VDR, 36 h unstim.
VDR, 36 h 1,25D
THP-1
VDR, 40 min unstim.
VDR, 40 min 1,25D
CD14
B
Chr 20:
23,100 kb
23,200 kb
23,300 kb
[0-450]
LS-180
VDR, 180 min EtOH
VDR, 180 min 1,25D
Lymphobl
VDR, 36 h unstim.
VDR, 36 h 1,25D
THP-1
VDR, 40 min unstim.
VDR, 40 min 1,25D
THBD

# Fig. S3

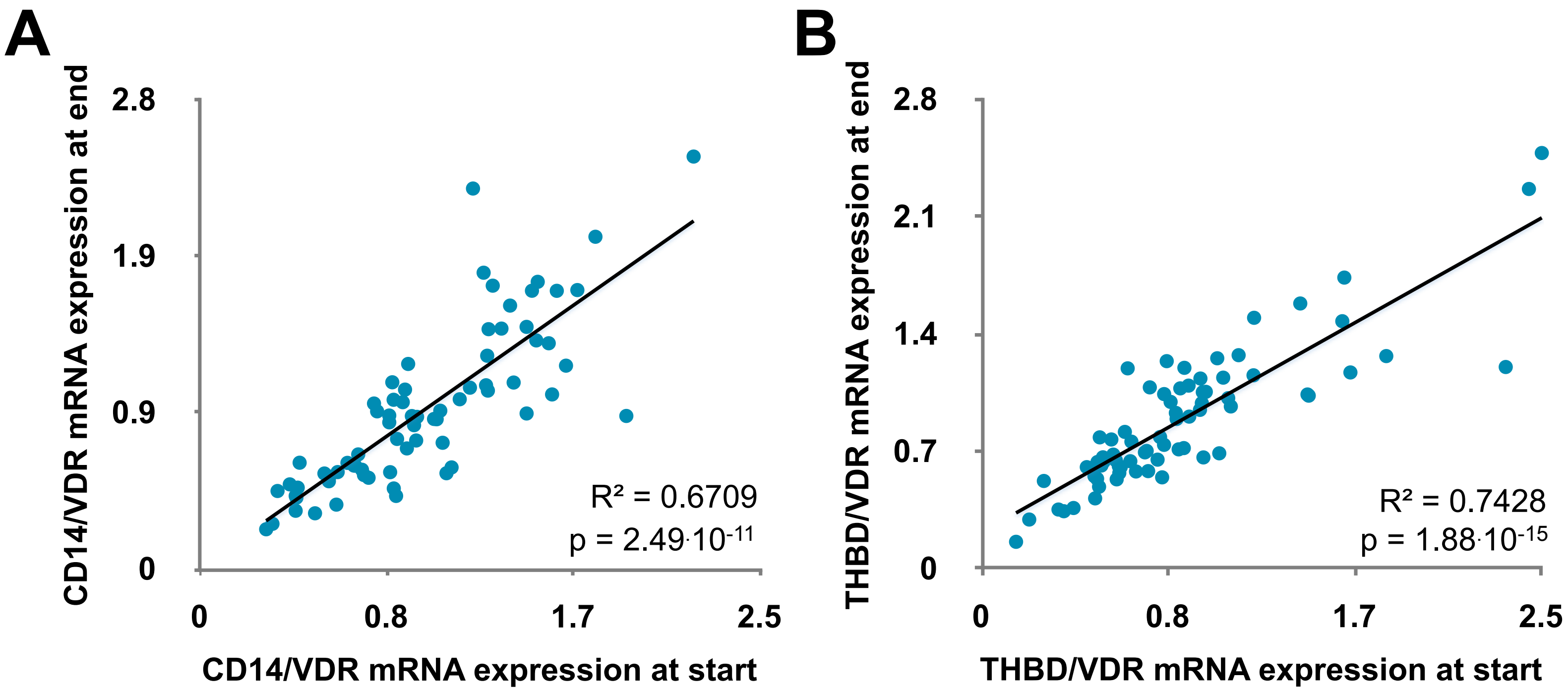
A
CD14/VDR mRNA expression at end
2.8
1.9
0.9
0
0
0.8
1.7
2.5
CD14/VDR mRNA expression at start
R² = 0.6709
p = 2.49·10-11
B
THBD/VDR mRNA expression at end
2.8
2.1
1.4
0.7
0
0
0.8
1.7
2.5
THBD/VDR mRNA expression at start
R² = 0.7428
p = 1.88·10-15
